# Effects of the addition of transcutaneous electrical stimulation to non-pharmacological measures in labor pain: study protocol for a randomized controlled trial

**DOI:** 10.1186/s13063-021-05969-0

**Published:** 2022-01-17

**Authors:** Naiara Toledo Dias, Patrícia Roberta Santos, Thais Alves Cândido, Rogério de Melo Costa Pinto, Ana Paula Magalhães Resende, Vanessa Santos Pereira-Baldon

**Affiliations:** 1 Health Sciences Post Graduation Program, Federal University of Uberlândia, Uberlândia, Brazil; 2 Health Sciences Post Graduation Program, Federal University of Goiás, Goiânia, Brazil; 3 Federal University of Uberlândia, R. Benjamin Constant, 1286 - Nossa Sra. Aparecida, Uberlândia, MG 38400-678 Brazil

**Keywords:** Pain, Labor, Non-pharmacological pain relief measures, Electrostimulation

## Abstract

**Background:**

Labor, although natural and physiological, is a period that can be marked by stress, pain, anxiety, suffering, fear, and anguish for a woman. Thus, non-pharmacological methods that reduce pain during labor are important to allow a better experience without the use of medications. Therefore, the aim of this study is to evaluate the effects of non-pharmacological pain relief methods, added or not to the application of transcutaneous electrical stimulation (TENS), on pain, satisfaction with the childbirth, duration of labor, and newborn conditions.

**Methods:**

This is a randomized controlled clinical trial, with a non-probabilistic convenience sample, composed of women in the first active stage of labor, admitted to a public institution. The parturients will be divided into 3 groups: group 1 (*n* = 36) composed of parturients who will have continuous support and will be encouraged to walk, adopting different positions with the use of the Swiss ball and receiving back massage for 30 min; group 2 (*n* = 36) composed of parturients who will also have continuous support and will be encouraged to walk, adopt different positions using the Swiss ball, and will receive the application of TENS for 30 min; and group 3 (*n* = 36) composed of parturients who will have continuous support and will be encouraged to walk, adopting different positions with the use of the Swiss ball, and will receive placebo TENS application for 30 min. The outcomes evaluated in the study will be pain intensity assessed by the visual analog scale of pain applied before, immediately after, and 30 min and 1 h after the interventions; Experience and Satisfaction with Childbirth Questionnaire (QESP) applied 12 to 24 h after delivery; and data regarding delivery (type of delivery, total duration of labor, and possible obstetric complications) and neonate (weight, height, possible complications, Apgar score in the first and fifth minutes).

**Discussion:**

With this research, it is expected to understand the effects of the intervention through TENS electrostimulation added to other non-pharmacological methods for pain management during labor.

**Trial registration:**

Brazilian Registry of Clinical Trials (REBEC) RBR-68kh6j. Registered on March 17, 2020

## Administrative information

Note: the numbers in curly brackets in this protocol refer to SPIRIT Checklist item numbers. The order of the items has been modified to group similar items (see http://www.equator-network.org/reporting-guidelines/spirit-2013-statement-defining-standard-protocol-items-for-clinical-trials/).

**Table:** 

Title {1}	Effects of the addition of transcutaneous electrical stimulation to non-pharmacological measures of relief during childbirth work: controlled randomized clinical study
Trial registration {2a and 2b}.	Brazilian Registry of Clinical Trials (REBEC), registration RBR-68kh6j
Protocol version {3}	Version 2 (September, 30, 2019)
Funding {4}	Coordenação de Aperfeiçoamento de Pessoal de Nível Superior (CAPES), Brazil – Finance Code 001. There is no direct funding to the project. CAPES provided necessary infrastructure for research to be developed. Thus, the financing of research-related expenses was the responsibility of the team of researchers involved in the project.
Author details {5a}	Federal University of Uberlândia, Brazil
Name and contact information for the trial sponsor {5b}	No sponsor
Role of sponsor {5c}	Not applicable

## Introduction

### Background and rationale {6a}

Labor, although natural and physiological, is a period of time that can be marked by stress, pain, anxiety, suffering, fear, and anguish for women [1]. Thus, the World Health Organization (WHO) recommends the use of non-pharmacological pain relief methods such as walking, kinesiotherapy or maternal mobility, exercise on a Swiss ball, massage, breathing exercises, relaxation techniques, and hot bath with the aim of reducing pain and promoting an active posture of the parturient, with greater autonomy for women [2].

Non-pharmacological methods of pain relief can be used alone or together with the multidisciplinary team (physiotherapists, nurses, nursing technicians, doctors, and doulas), depending on the parturient’s choice and the hospital’s infrastructure [3–6]. Some studies have demonstrated the effectiveness of walking and massage in reducing pain and increasing pain tolerance during labor [7–9]. Besides that, studies with other methods such as breathing and relaxation techniques, maternal mobility, Swiss ball exercises, and hot bath have observed a decrease in the use of medications, a decrease in anxiety and stress, a decrease in the duration of the active phase of labor, and greater body perception of the parturient [10–12].

A non-pharmacological method that has been investigated is the use of transcutaneous electrical stimulation (TENS). It is an equipment used for pain relief, and studies have analyzed its effect during labor [13, 14]. There are two theories that explain the action of TENS in reducing pain. The first is the theory proposed by Melzack and Wall in 1965 that explains the action of TENS through the gate control theory. Thus, TENS provides electrical stimulation through the skin, through sensory stimuli that will carry information to the brain through afferent fibers to the dorsal horn of the Aß medulla [15, 16]. Therefore, there is a blockage of pain impulses to the brain, as this fiber transmits the information faster than the fibers responsible for transmitting pain [15, 16]. Another theory is explained by the release of endogenous opioids by the brain, such as beta-endorphins, which have an analgesic effect [15, 16].

For the use of TENS during labor, the electrodes are placed at the level of the T10–L1 and S2–S4 vertebrae [14, 17]. During application, the parturient reports tingling or tickling, without causing muscle contraction or pain. Studies have shown the safety of the method for the mother and fetus and positive effects during labor such as reduced pain, anxiety, labor duration, and use of complementary analgesia and improved satisfaction [13–19].

Despite the number of studies on the use of TENS in labor and its wide use in clinical practices, systematic reviews on the subject show the low quality of studies so far [18, 20]. Thuvarakan et al. [20], in a meta-analysis, found only a small efficacy of TENS in reducing pain intensity during labor. It is not possible to say whether the results were affected by the low quality of the studies or not.

Furthermore, the reality of clinical practice is the use of non-pharmacological methods of pain relief together with an attempt to provide greater relief to women. However, the current scientific evidence comes mainly from studies that evaluated the isolated effects of the techniques [12]. Therefore, it is important to analyze the possible additional effect of using TENS in addition to other non-pharmacological pain relief methods through a clinical trial with greater methodological control. These results can contribute to the clinical practice of the physiotherapists in obstetrics, justifying or not the investment in equipment in maternity hospitals.

### Objectives {7}

The aim of this randomized clinical trial is to verify the effects of non-pharmacological methods of pain relief added to the application of TENS on pain, satisfaction with childbirth, duration of labor, and the birth conditions of newborns.

### Trial design {8}

This is a single-blind randomized controlled superiority trial to compare the three groups randomized in parallel (1:1:1). This article has been written in accordance with the Standard Protocol Items: Recommendations for Interventional Trials (SPIRIT) guidelines.

## Methods: participants, interventions, and outcomes

### Study setting {9}

The trial will be carried out at the Hospital Municipal Modesto de Carvalho, in the city Itumbiara, GO, Brazil.

### Eligibility criteria {10}

Patients eligible for inclusion in the trial must meet all the following criteria:

- Pregnant women in active labor
- Low-risk pregnancies
- Women with a gestational age of 37–42 weeks
- Gestation with a single fetus in the cephalic position

### Exclusion criteria

The following are the exclusion criteria:

- Having a wound or inflammation in the cutaneous areas of application of the TENS electrodes
- Presence of a pacemaker
- Inability to understand verbal commands

### Who will take informed consent? {26a}

A signed informed consent form will be obtained from each patient prior to their participation in the study. The evaluator will acquire consent during hospitalization. The evaluator will explain all steps of the study, and the volunteers who agree to participate will sign the term.

### Additional consent provisions for the collection and use of participant data and biological specimens {26b}

Not applicable. There will be no biological sample collection in this study.

### Interventions

#### Explanation for the choice of comparators {6b}

In clinical practice, non-pharmacological methods of pain relief are used together, in an attempt to promote better results. However, no studies were found in the literature that verified, through the comparison of non-pharmacological methods of pain relief already recommended by the WHO and added to the application of TENS, whether such a resource would bring a potential reduction in pain in parturients during labor. Therefore, it was decided to compare non-pharmacological methods of pain relief with the same methods added to the use of TENS and placebo.

#### Intervention description {11a}

The intervention of the three groups will take place during the first active phase of labor, from admission, which occurs when the parturient has 4 cm of cervical dilation, until the beginning of the expulsive period. The three groups will receive continuous support from professionals, encouraging walking and the adoption of vertical postures (standard intervention). In addition, parturients in group 1 will be positioned on a Swiss ball to receive a massage in the dorsal region for 30 min. Classic massage movements will be used, such as superficial sliding, deep sliding, kneading, friction, and rolling. Almond oil will be used to facilitate manual maneuvers.

**Fig. 1:**
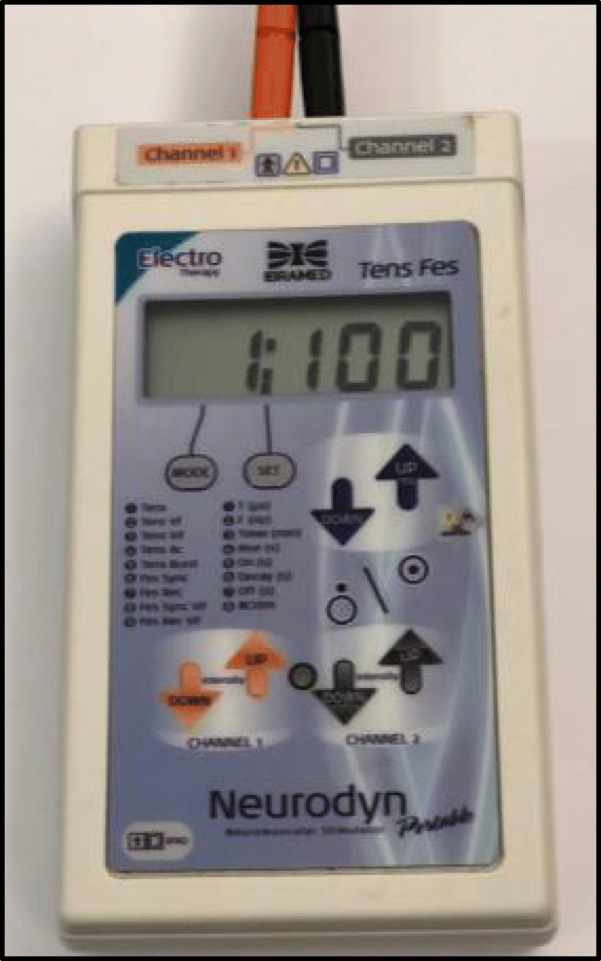
Image of electrode placement in the thoracolumbar region

**Fig. 2:**
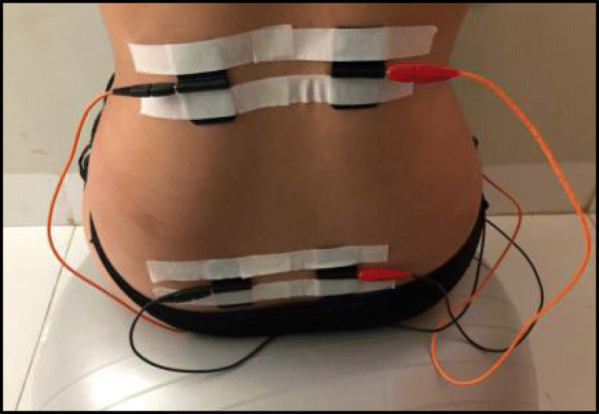
Image of the portable TENS equipment

Women in group 2 will undergo a standard intervention plus an intervention using TENS electrostimulation using the portable Neurodyn equipment (IBRAMED-Indústria Brasileira de Equipamentos Médicos EIRELI, Brazil). The parturients will be positioned seated on the Swiss ball, and four silicone electrodes will be fixed with masking tape in the thoraco-lumbar region, at the levels of T10–L1 and S2–S4. A frequency of 100 Hz and a pulse width of 100μs will be used, with intensity depending on the sensitivity of each patient for 30 min [17] (Figs. 1 and 2. Women in group 3 will undergo standard intervention and placebo intervention. They will be positioned and have electrodes attached in a similar way to group 2. However, the equipment will remain with the intensity button deactivated, without current flow (Figs. 1 and 2).

#### Criteria for discontinuing or modifying allocated interventions {11b}

The criteria for discontinuation are any medical complications during delivery or at the request of the participant. The women can also withdraw their consent prior to the publication of the results.

#### Strategies to improve adherence to interventions {11c}

As this is an intervention that happens only during labor, the researchers will explain in detail the procedure for participating in the project and the need for data collection after delivery.

#### Relevant concomitant care permitted or prohibited during the trial {11d}

During the protocol, oral and intravenous medications indicated by the physician are allowed, except for analgesics. Analgesic medications are not part of the hospital protocol, following government recommendations, so there will be no harm to the participants. Maternal mobility, bathing, and total freedom for the parturients in labor are allowed, as recommended by the WHO.

#### Provisios for posttrial care {30}

Researchers are responsible for any harm that occurs as a result of the study. Thus, researchers will provide participants with the health care needs that may arise as a direct consequence of trial participation.

### Outcomes {12}

#### Primary outcome measure

The primary outcome measure is change in the visual analog scale (VAS) from baseline to immediately after the intervention. The VAS is a simple one-dimensional instrument, used worldwide to assess pain intensity. It is characterized by a 10-cm-long horizontal line, where 0 represents no pain and 10 the worst imaginable or severe pain. They will be evaluated before the intervention and immediately after the intervention. All measurements will be performed in the period between uterine contractions.

#### Secondary outcome measures

The secondary outcomes include change in the visual analog scale (VAS) from baseline to 30 min and 1 h after the intervention and in the immediate postpartum period (12–24 h after labor).

The participant’s satisfaction with childbirth will also be evaluated using the Experience and Satisfaction with Childbirth Questionnaire (QESP). This is a self-report questionnaire, with a total of 104 questions, which refer to the expectations, experience, satisfaction, and quality of the woman’s experience in relation to labor, delivery, and immediate postpartum. It consists of questions divided into eight subscales: (1) conditions and care provided, (2) positive experience, (3) negative experience, (4) relaxation, (5) social support, (6) partner’s support, (7) concerns, and (8) postpartum. The higher the score obtained in each of the subscales, the more positive is the perception of women in the dimension assessed by the subscale. The administration time for this questionnaire is approximately 30 min [21]. The questionnaire will be applied in the immediate postpartum period (12–24 h after delivery).

After delivery, information on the type of delivery, total duration of delivery, and possible obstetric complications will be collected from the participants’ medical records. Newborn data will also be collected from medical records: weight, height, possible complications, and Apgar score in the first and fifth minutes.

### Participant timeline {13}

Initially, volunteers will be numbered to avoid the risk of being identified. Soon after admission to the hospital, they will answer the standard anamnesis composed of questions about gynecological, obstetric, and health history, lasting approximately 10 min.

Before starting the intervention, the volunteer will be asked to mark the intensity of her pain at the moment on the VAS line. Immediately after and 30 min and 1 h after the interventions with the use of massage, TENS, or placebo, the request for pain intensity indication will be repeated.

After 12 to 24 h after the end of delivery, the volunteer will be visited by the researcher in the rooming-in and will be instructed to answer the intensity of pain in the VAS at this time, in addition to answering the QESP questionnaire. Then, birth and newborn data will be collected from the medical records.

**Fig. 3:**
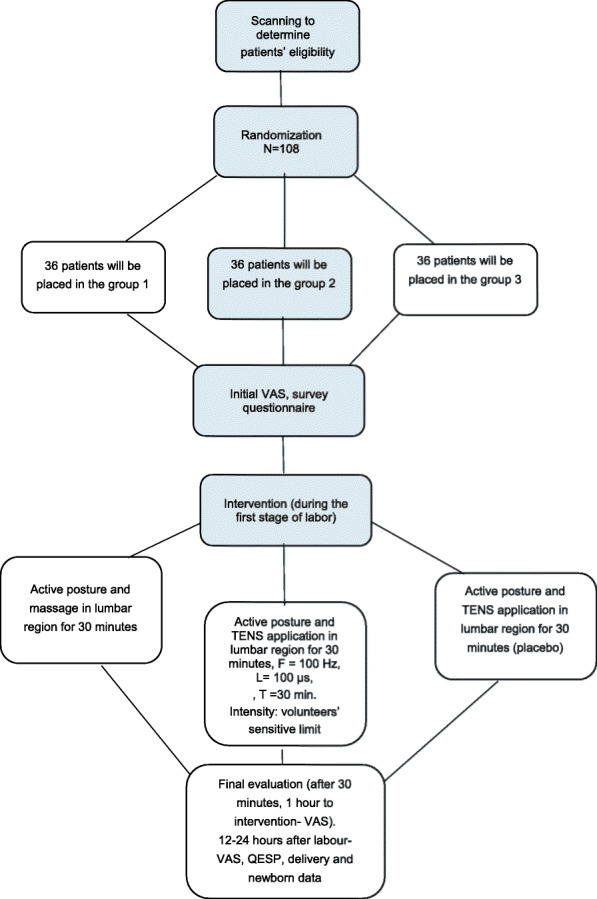
Flow diagram of the trial design

The schematic diagram of the participant’s timeline can be seen in Fig. 3.

### Sample size {14}

Based on a previous study by Báez-Suárez et al. [14], considering a difference between groups of 1.5 in VAS and a standard deviation of 1.61, considering an alpha of 0.05 and the power of the test of 0.80, the minimum sample size in each group is 36 participants.

### Recruitment {15}

Participants will be recruited at the Modesto de Carvalho Municipal Hospital, in the city Itumbiara, GO, Brazil. This hospital has an average of 67 births per month, with a rate of vaginal birth around 43.51%. No advertising is allowed for recruitment, and no inducements will be given to recruit patients into the study.

## Assignment of interventions: allocation

### Sequence generation {16a}

Participants will be randomized in a ratio of 1:1:1 to the three groups: group 1 (standard intervention and massage in lumbar region for 30 min), group 2 (standard intervention and TENS application in lumbar region), and group 3 (standard intervention and TENS placebo application in lumbar region for 30 min), using computer-generated random numbers.

### Concealment mechanism {16b}

A researcher not involved in the data collection will assign the groups using the sealed envelope method. The opaque envelopes will be numbered and kept locked, available only to the study researchers.

### Implementation {16c}

The allocation list will be generated by the mathematician who is part of the research team. All patients who give consent for participation and who meet the inclusion criteria will be randomized. After the participant has been included, the researcher responsible for the interventions will open the envelope with the lowest number available to determine which group the participant belongs to.

## Assignment of interventions: blinding

### Who will be blinded? {17a}

The researcher in charge of the interventions to be applied to the participants will be blind to the assessments. The researcher responsible for the evaluations and the researcher responsible for data processing will be blind for the treatment allocation. Parturients will not be totally blind to the procedure due to the difficulties imposed by the differences between techniques, but participants in the placebo group will not have information about the functioning of the equipment.

### Procedure for unblinding if needed {17b}

The loss of blinding should not be carried out unless there is a threat to the participant’s safety as a consequence of the study procedures.

## Data collection and management

### Plans for assessment and collection of outcomes {18a}

A trained researcher will perform all assessments during labor and after delivery. The VAS is a simple one-dimensional instrument, used worldwide to assess pain intensity. Studies demonstrate that the VAS is valid, reliable, and adequate to measure pain both in the clinical situation and in the evaluation of obstetric pain, in addition to being reliable to measure acute pain [22–24].

To assess the birth experience, the Childbirth Experience and Satisfaction Questionnaire (QESP) was chosen. It was built and validated in Portuguese and has good internal consistency and a test-retest fidelity index [21].

### Plans to promote participant retention and complete follow-up {18b}

Participants will be followed during their usual stay after delivery, which lasts 48 h in this hospital.

### Data management {19}

Following the institution’s protocols, all data will be considered anonymous, and participants will be identified by numbers. All source documents will be stored in locked file cabinets with secure and limited access. The data will be transferred to a secure online data cloud through double-checking between researchers. Only the research group has an individual password to access the data.

### Confidentiality {27}

The study will be conducted in accordance with the Brazilian rules and regulations. All data generated in this study will remain confidential. Only the research team has access to the study data. Access to data will only be provided in the event of audits or regulatory regulation by the institution.

### Plans for collection, laboratory evaluation, and storage of biological specimens for genetic or molecular analysis in this trial/future use {33}

Not applicable as no biological samples will be collected.

## Statistical methods

### Statistical methods for primary and secondary outcomes {20a}

Obstetric, gynecological, sociodemographic data, among others, will be compiled and collected using the statistical program Statistical Package for Social Sciences (SPSS Statistics version 23) and tabulated in the Excel program. The Shapiro-Wilk test will be applied to test the normality of the data, whether the data follow a normal distribution or follow a non-normal distribution.

The data will be presented to the normality of these, according to the quantitative variables that are normally distributed and continuously will be presented as mean (standard deviation). Continuous quantitative variables without normal distribution will be presented in median (interquartile range). Categorical variables will be assessed as frequencies and percentages.

For parametric data, a comparison between the groups will be carried out using the analysis of variance (ANOVA) test. Significance values lower than 0.05, at the 95% confidence interval, will be interpreted as statistically significant. The clinical relevance of the recorded values will be confirmed through effect size calculations (Cohen *d*) based on significant differences. The following effects will be taken into account: 0.00 to 0.49, low; 0.50 to 0.79, medium; and above 0.80, 312 high [25].

### Interim analyses {21b}

No interim analysis will be performed.

### Methods for additional analyses (e.g., subgroup analyses) {20b}

None was planned.

### Methods in analysis to handle protocol non-adherence and any statistical methods to handle missing data {20c}

Any missing data and the reason for the missing data will be described for each group. If necessary, a multiple imputation model will be applied.

### Plans to give access to the full protocol, participant level-data, and statistical code {31c}

The datasets analyzed during the study will be available from the corresponding author upon reasonable request.

### Oversight and monitoring

#### Composition of the coordinating center and trial steering committee {5d}

The researchers involved in the study (see on title page) form the coordinating center which is responsible for study coordination, monitoring, data acquisition and management, and statistical analysis.

#### Composition of the data monitoring committee, its role, and reporting structure {21a}

This study will not have a data monitoring committee, as it is a short-term trial with minimal known risks.

#### Adverse event reporting and harm {22}

This study involves minimal known risks, but the researcher responsible for the evaluation will question the participants about possible adverse effects. Any harm detected will be reported to the institution’s research ethics committee.

#### Frequency and plans for auditing trial conduct {23}

No audits are planned because this trial is academic.

### Plans for communicating important protocol amendments to relevant parties (e.g., trial participants, ethical committees) {25}

According to the national regulations, major modifications of the protocol require a formal amendment to the protocol and must be approved by the institution’s research ethics committee and modified in the Brazilian Registry of Clinical Trials.

### Dissemination plans {31a}

The trial results will be submitted for publication in relevant journals and presented at conferences in the field of physiotherapy, gynecology, and obstetrics. In addition, the results will be published on the university’s social network in accessible language so that they are known by the population.

## Discussion

The feeling of pain during labor is feared by pregnant women and can be one of the factors that lead women to move away from the decision for vaginal birth. Thus, health professionals look for ways to relieve pain and improve the woman’s experience of childbirth. Non-pharmacological methods of pain relief are widely used during childbirth because they have benefits without side effects or contraindications [26].

Despite its widespread use, there is a lack of scientific evidence to support the use of some techniques, including TENS. Thuvarakan et al. [20], in a meta-analysis, observed that the studies have difficulty in a detailed description regarding randomization with the most appropriate methods, such as the allocation, masking, and blinding of the study. Thus, it was detected that many studies had limitations that compromise the quality of the studies and, therefore, their conclusions.

With this research, it is expected to contribute to the understanding of the effects of the intervention through TENS, in addition to other non-pharmacological pain relief measures in parturients. Furthermore, with the dissemination of the conclusions obtained in this study through publication in scientific journals and events in the area, health professionals will have important information for conducting pain management through non-pharmacological pain relief measures during labor.

## Trials status

The study was registered at the Brazilian Registry of Clinical Trials (REBEC) (number RBR-68kh6j) on March 17, 2020. Recruitment for the study began on April 01, 2020, and the planned recruitment completion date is February 28, 2022.

## Abbreviations

TENS: Estimulação Elétrica Transcutânea
ANOVA: Analysis of variance
*F*: Frequency
REBEC: Brazilian Registry of Clinical Trials
SPIRIT: Standard Protocol Items: Recommendations for Interventional Trials
SPSS: Statistical Package for Social Sciences
*T*: Pulse length
VAS: Visual analog scale
